# Old London Bridge

**Published:** 1885-07

**Authors:** 


### Old London Bridge.

London bridge, had it been destroyed recently by the dynamite plotters, would have left a gap in metropolitan arrangements not easily to be filled up. This, however, could not have been said of the old London bridge. The venerable structure was 915 feet in length. The street that covered it consisted, before the houses fell to decay, of lofty edifices, built with some attention to exterior regularity. It was twenty feet wide, and the buildings on either side about twenty-six feet in depth. Across the middle of the street ran several lofty arches, extending from side to side; the bottom part of each arch terminating at the first story, and the upper part reaching near the tops of the houses. They were designed to prevent the buildings from giving way, and were therefore formed of strong timber bolted in the corresponding woodwork of the house that flanked them. Thus the street on the bridge had nothing to distinguish it from any narrow street in the city but the high arches described and three openings guarded with iron rails, which afforded a view of the river.

But the appearance from the water, it is stated, "baffled all description" and displayed a strange example of curious deformity. Nineteen unequilaterial arches of different heights and breadths, with sterlings increased to a monstrous size by frequent repairs, served to support a range of houses as irregular as themselves; "the back part of which, broken by hanging and irregular projections, offered a very disgusting object," while many of the buildings overhung the arches so as to hide the upper part of them, and seemed to lean in such a manner "as to fill the beholder with equal amazement and horror." Such was London bridge in olden times.—*St. James' Gazette.*

RATTLESNAKE bite was the cause of death in New York city recently. Some years ago, before a commission of medical men in Philadelphia, a man, for a money consideration, allowed himself to be bitten for the purpose of proving the value of *plantago major* (common plantain), and the result was satisfactory.

FATAL sunstroke never occurs in a person who abstains from alcoholics.—*Dr. G. R. Patton, Minnesota.*

